# Single versus multiple human-equivalent doses of C. parvum in mice: neutralization of the anti-metastatic effect.

**DOI:** 10.1038/bjc.1980.64

**Published:** 1980-03

**Authors:** H. D. Mitcheson, T. E. Sadler, J. E. Castro

## Abstract

The murine dose of i.v. C. parvum (466 microgram) was compared with a single, low, human-equivalent dose of 70 microgram and with repeated weekly low doses. All treatments increased the antibody titre against C. parvum (CP). However, repeated doses stimulated a much higher titre than single doses. In all treated animals spleen weight peaked at 2 weeks and then fell. A single low dose caused a 3-fold increase, a single high dose or multiple low doses a 6-fold increase. Liver weight changes followed a similar pattern. Hepatosplenomegaly was prolonged by multiple doses. The effects of these treatments on Lewis tumour metastases were studied. A single high dose and a single low dose on the day of tumour implantation (Day 0) were equally effective at inhibiting pulmonary metastases. Repeated low doses starting on Day 0 were no more effective than a single dose. The effect of CP on survival after primary-tumour excision on Day 10 was observed. Low dose CP on Day 7 doubled the harmonic mean of survival time. Repeated doses were no more effective than a single dose. Low-dose prophylaxis up to 2 weeks before tumour significantly inhibited metastases. However, when repeated low-dose prophylaxis was combined with a single low dose on Day 0, the anti-metastatic effect was abrogated. This neutralization of the anti-metastatic effect of CP given on Day 0 was found to persist after a 13-week treatment-free interval. Possible mechanisms for this phenomenon are discussed.


*Br. J. Cancer* (1980) **41**, 407

### SINGLE *VERSUS* MULTIPLE HUMAN-EQUIVALENT DOSES OF *C. PARVUM* IN MICE: NEUTRALIZATION OF THE ANTI-METASTATIC EFFECT

H. D. MITCHESON, T. E. SADLER AND J. E. CASTRO

*From the Department of Surgery, Royal Postgraduate Medical School, Du Cane Road, London*

Received 6 September 1979 Accepted 21 November 1979

**Summary.—The murine dose of i.v. *C. parvum* (466 μg) was compared with a single, low, human-equivalent dose of 70 μg and with repeated weekly low doses. All treatments increased the antibody titre against *C. parvum* (CP). However, repeated doses stimulated a much higher titre than single doses. In all treated animals spleen weight peaked at 2 weeks and then fell. A single low dose caused a 3-fold increase, a single high dose or multiple low doses a 6-fold increase. Liver weight changes followed a similar pattern. Hepatosplenomegaly was prolonged by multiple doses.**

**The effects of these treatments on Lewis tumour metastases were studied. A single high dose and a single low dose on the day of tumour implantation (Day 0) were equally effective at inhibiting pulmonary metastases. Repeated low doses starting on Day 0 were no more effective than a single dose. The effect of CP on survival after primary-tumour excision on Day 10 was observed. Low dose CP on Day 7 doubled the harmonic mean of survival time. Repeated doses were no more effective than a single dose.**

**Low-dose prophylaxis up to 2 weeks before tumour significantly inhibited metastases. However, when repeated low-dose prophylaxis was combined with a single low dose on Day 0, the anti-metastatic effect was abrogated. This neutralization of the anti-metastatic effect of CP given on Day 0 was found to persist after a 13-week treatment-free interval. Possible mechanisms for this phenomenon are discussed.**

SYSTEMICALLY INJECTED *C. parvum* (CP) has considerable anti-tumour properties in rodents (Halpern *et al.*, 1966; Woodruff & Boak, 1966; Smith & Scott, 1972; Proctor *et al.*, 1973; Sadler & Castro, 1976). It is a potent stimulant of the reticulo-endothelial system (RES) (Halpern *et al.*, 1963; Adlam & Scott, 1973) and non-specific activation of macrophages is generally regarded as mediating the anti-tumour effect (reviewed by Milas & Scott, 1978). Cancer patients have been treated with systemic CP (Israel *et al.*, 1975; Takita & Moayeri, 1976; Sarna *et al.*, 1977) but there is still no convincing evidence of a beneficial antitumour response. This difference between man and rodents may be due to the dose of the vaccine since, owing to the severity of the side-effects, the human dose, when related to surface area, is much lower than the murine dose (Scott & Warner, 1976).

The aim of this study was to determine, in mice, the best treatment regimen for cancer using CP. We compared our usual mouse dose (a single injection of 466 μg) with a single low, human-equivalent dose (70 μg) and with repeated human-equivalent doses.

### MATERIALS AND METHODS

*Mice.*—Age-matched syngeneic female C57BL mice (Olac) were used.

C. parvum.—A heat-killed suspension of *C. parvum* (Wellcome, strain CN6134, 7 mg dry weight/ml) was used. The high dose was 466 μg and the low or human-equivalent dose was 70 μg. This was calculated from our clinical dose of 10 mg/m$^2$ (Mitcheson & Castro, 1978) relating the surface area of a

20g mouse to a 70kg human. Repeated treatments were given weekly. The usual route of administration was i.v. but in certain experiments, designed to determine the best route, i.p. and s.c. injections were used.

*Toxicity*.—Toxicity was monitored by observing animals for 4 h after injection and recording the side-effects.

C. parvum *antibody*.—The serum was stored at −18°C and antibodies to CP measured by passive agglutination. Doubling dilutions of serum were made with phosphate-buffered saline to a total volume of 25 μl in each well of a microtitre plate. 25 μl of a 0·7mg/ml CP suspension was added to each well. Positive and negative controls were included in the test. The mixtures were incubated for 2 h at 37°C and then at 4°C for 48 h. Agglutination was observed and the antibody titres expressed as powers of 2 ($\log_2$).

*Organ weights and histology*.—Four mice were removed from each group at weekly intervals, weighed, anaesthetized with ether and exsanguinated from the heart. The liver, spleen and thymus were removed and immediately weighed. These organs, together with lung and kidney, were fixed in formal saline and histological appearances noted after examination of sections stained with haematoxylin and eosin.

*Tumour*.—Lewis lung carcinoma, which originated spontaneously as a carcinoma of the lung of a C57/BL mouse at the Wistar Institute in 1951 (Sugiura & Stock, 1955) was implanted s.c. as a 0·1ml homogenate in the lower flank. It is a rapidly growing epidermoid carcinoma which, when implanted s.c., metastasizes to the lungs (Simpson-Herren *et al.*, 1974). Cells are released from the primary tumour 6 days after implantation (James & Salsbury, 1974) and macroscopic metastases are easily visible 21 days after implantation.

*Anti-tumour effects*.—Two experimental systems were used. In the first the tumour was inoculated s.c. on Day 0 and the growth of the primary tumour monitored by measuring 2 diameters twice weekly and calculating the mean diameter. The mice were killed on Day 21 and macroscopic lung metastases counted after staining the lungs by inflation with Indian ink (Wexler, 1966). In the second the tumour was implanted on Day 0 and subsequently excised on Day 10 (Sadler & Castro, 1976). The survival of the mice after tumour excision was noted.

*Statistics*.—Results were compared using Student's *t* test. Group survival was expressed as the harmonic mean survival time.

### RESULTS

### *Biological effects*

*Toxicity*.—Mice given a high dose of CP became very ill for several hours with erect fur, dyspnoea and a slow, staggering gait. The low dose had little adverse effect. All mice had a decrease in body weight which lasted 2–3 weeks and this was greater in mice receiving the larger dose.

*Antibody titre*.—Antibody titres expressed as the power of 2 are shown in Fig. 1. Untreated mice had a low natural titre. A single high dose increased this titre progressively to 12 at 6 weeks and a single dose to 11. Repeated low doses stimulated a more rapid and pronounced rise to 26.

*Organ weights*.—Spleen weight after CP

FIG. 1.—Antibody titres in C57BL mice at weekly intervals after a single injection of 70 μg *C. parvum* (● .... ●), of 466 μg (●——●) and during the course of 6 weekly injections of 70μg (● - - - ●). Each point represents the mean from 4 mice and the bar denotes s.e.

FIG. 2.—Spleen weights in C57BL mice at weekly intervals after a single injection of 70 μg *C. parvum* (● . . . . ●), of 466 μg (●——●) and during the course of 6 weekly injections of 70 μg (● - - - ●). Each point represents the mean from 4 mice and the bar denotes s.e.

FIG. 3.—Liver weights in C57BL mice at weekly intervals after a single injection of 70 μg *C. parvum* (● . . . . ●), of 466 μg (●——●) and during the course of 6 weekly injections of 70 μg (● - - - ●). Each point represents the mean from 4 mice and the bar denotes s.e.

is shown in Fig. 2. In all treated mice, spleen weight peaked at 2 weeks and then fell. A single low dose caused a 3-fold increase in spleen weight. A single high dose or multiple low doses caused a 6-fold increase. This splenomegaly was prolonged by multiple doses. Liver weight is shown in Fig. 3 and the changes follow a similar pattern to that of the spleen. Thymus weight is shown in Fig. 4. Cortical atrophy of the thymus occurred and followed an inverse pattern to the alterations of spleen and liver weights.

In mice receiving repeated injections, liver and spleen weights were greatest 2 weeks after the first injection and then fell, despite further injections. In a separate experiment to determine when the organs could be restimulated after an initial low dose of CP, different groups of mice received a second low dose 2, 4, 6, 8, 10 or 12 weeks later. Mice were killed 2 weeks after the second injection and their liver and spleen weights compared with organ weights 2 weeks after a first injection. The development of hepatospleno-

FIG. 4.—Thymus weights in C57BL mice at weekly intervals after a single injection of 70 μg *C. parvum* (● . . . ●), of 466 μg (●——●) and during the course of 6 weekly injections of 70 μg (● - - - ●). Each point represents the mean from 4 mice and the bar denotes s.e.

megaly was impaired in mice which received a second injection 2 weeks after the first, but was not impaired in those receiving the second injection after 4 or more weeks.

*Histology.*—A single high dose caused thrombosis in hepatic, pulmonary and splenic vessels, with consequent hepatic necrosis. There was considerable recovery after 4 weeks. A single low dose caused a similar but much less severe pathology. Multiple low doses had the same effects as a single low dose and no fresh pathology occurred after the second or subsequent injections. Considerable recovery was observed 4 weeks after the first injection.

*Anti-tumour effects*

*Single doses.*—Table I shows the effect on tumour metastasis of various single doses of CP given on the same day as Lewis tumour (Day 0). A single high dose (466 μg) and a single human-equivalent dose (70 μg) were equally effective in significantly reducing pulmonary metastasis. The lowest dose to inhibit tumour metastasis was 35 μg.

TABLE I.—*The effect on Lewis tumour metastasis of various i.v. doses of CP on Day* 0

| Dose of CP on Day 0 (μg) | No. of mice | Mean no. of pulmonary metastases ± s.d. | *P* (*vs* control) |
|---|---|---|---|
| — | 7 | 16 ± 13 | |
| 466 | 9 | 3 ± 6 | < 0·002 |
| 140 | 8 | 1 ± 1 | < 0·01 |
| 70 | 9 | 3 ± 3 | < 0·01 |
| 35 | 8 | 1 ± 1 | < 0·01 |
| 17·5 | 7 | 12 ± 14 | not sig. |

*Single* vs *repeated doses.*—The anti-metastatic effect of a single low dose (70 μg) given on the day of tumour implantation was compared with that of repeated low doses. Two groups of mice received repeated doses: one on Days 0 and 7, and the other on Days 0, 7 and 14. The animals were killed on Day 21. The primary tumour growth and the mean number of pulmonary metastases for each

TABLE II.—*The effect on primary Lewis tumour and its metastases of a single i.v. low dose of* 70 *μg CP and repeated low doses*

| Treatment on Days | No. of mice | Mean diam (mm) of primary tumour on Day 21 ± s.d. | *P* (*vs.* control) | Mean no. of pulmonary metastases ± s.d. | *P* (*vs.* control) |
|---|---|---|---|---|---|
| — | 15 | 23·9 ± 4·4 | | 20 ± 14 | |
| 0 | 16 | 19·8 ± 2·9 | < 0·01 | 4 ± 4 | < 0·001 |
| 0, 7 | 17 | 19·6 ± 2·8 | < 0·01 | 3 ± 3 | < 0·001 |
| 0, 7, 14 | 17 | 18·1 ± 2·4 | < 0·001 | 2 ± 2 | < 0·001 |

group are shown in Table II. All CP treatments inhibited the growth of the pulmonary tumour and significantly reduced the number of pulmonary metastases. Repeated doses were no more effective than a single dose.

*Survival: single* vs *repeated doses.*—In this experiment the tumour was inoculated on Day 0 and excised on Day 10. The control group received no other treatment. Mice received low dose CP as a single injection on Day 7 or Day 13, or repeated weekly injections starting on Day 7 or Day 13.

TABLE III.—*The effect of CP regimens on harmonic mean survival after tumour excision on Day* 10

| Treatment on Days | No. of mice | Harmonic mean survival time after tumour excision (days) |
|---|---|---|
| — | 10 | 11·0 |
| 7 | 9 | 21·7 |
| 7, 14, 21, 28, 35, 42 | 9 | 22·4 |
| 13 | 10 | 8·5 |
| 13, 20, 27 | 10 | 13·2 |

Table III shows the harmonic mean survival time after tumour excision. The control group had a harmonic mean survival of 11·0 days. Survival was twice as long in those groups that received CP on Day 7, either as a single dose or as repeated doses starting on that day. Treatment on Day 13, either as a single dose or as repeated doses starting on that day, was ineffective.

TABLE IV.—*The effect on primary Lewis tumour and its metastases of prophylactic single i.v. injections of CP*

| Dose of CP (μg) | On Day | No. of mice | Mean diam. (mm) of primary tumour on Day 21 ±s.d. | P (cf. control) | Mean no. of pulmonary metastases ±s.d. | P (cf) control) |
|---|---|---|---|---|---|---|
| — | | 10 | 19·7±2·1 | | 11±12 | |
| 70 | 0 | 8 | 16·5±2·6 | <0·02 | 1±1 | <0·1 |
| 70 | −7 | 8 | 14·9±2·4 | <0·001 | 1±1 | <0·1 |
| 70 | −14 | 9 | 19·0±2·4 | — | 3±5 | <0·1 |
| 70 | −21 | 9 | 17·8±3·3 | — | 6±7 | — |
| 466 | −21 | 10 | 19·0±1·9 | — | 5±5 | — |

*Single-dose prophylaxis.*—CP was given at intervals up to 3 weeks before the tumour (Table IV). Treatment one week before tumour significantly inhibited primary tumour growth and significantly reduced the number of pulmonary metastases and was as effective as treatment on the day of tumour implantation. Earlier pre-treatment did not inhibit primary tumour growth, though the number of pulmonary metastases was significantly reduced by treatment 2 weeks before tumour. Treatment 3 weeks before tumour was ineffective.

*Combination of low-dose prophylaxis with a further dose at tumour inoculation.*—Repeated low-dose prophylaxis was combined with an additional low dose on Day 0 (Table V). Three groups received prophylaxis, the first group on Days −21, −14 and −7, the second on Days −14 and −7 and the third on Day −7. A separate group received CP only on Day 0. The growth of the primary tumour was similar in all groups. The number of pulmonary metastases was significantly reduced in mice receiving CP only on Day 0. However, in mice which received prophylaxis and CP on Day 0 there was no inhibition of metastases.

TABLE V.—*The effect on primary Lewis tumour and its metastases of low dose (70 μg) i.v. CP prophylaxis combined with a further low dose on Day 0*

| CP on Day | No. of mice | Mean diam. (mm) of primary tumour on Day 21 ±s.d. | P (cf. control) | Mean no. of pulmonary metastases ±s.d. | P (cf. control) |
|---|---|---|---|---|---|
| — | 14 | 24·8±2·8 | | 44±13 | |
| 0 | 14 | 23·7±2·5 | — | 16±12 | <0·001 |
| −7, 0 | 20 | 25·7±1·9 | — | 39±14 | — |
| −14, −7, 0 | 16 | 25·7±1·7 | — | 53±22 | — |
| −21, −14, −7, 0 | 18 | 24·8±2·0 | — | 44±14 | — |

*Low-dose prophylaxis followed by a 13-week treatment-free interval.*—The preceding experiment was repeated leaving a 13-week treatment-free interval between prophylaxis and tumour implantation (Table VI). A similar result occurred; the anti-metastatic effect of a single low dose at the time of tumour implantation was abrogated by prophylaxis.

TABLE VI.—*The effect on primary Lewis tumour and its metastases of low dose i.v. CP prophylaxis, followed by a 13-week treatment-free interval, before tumour inoculation accompanied by a further low dose*

| CP on Day | No. of mice | Mean diam. (mm) of primary tumour on Day 21 | P (cf. control | Mean no. of pulmonary metastases ±s.d. | P (cf. control) |
|---|---|---|---|---|---|
| — | 9 | 19·5±2·0 | | 22±10 | |
| 0 | 9 | 18·8±1·8 | — | 6±4 | <0·001 |
| −90, 0 | 7 | 17·7±2·4 | — | 16±21 | — |
| −97, −90, 0 | 8 | 20·1±1·5 | — | 26±18 | — |
| −104, −97, −90, 0 | 7 | 18·9±1·5 | — | 16±11 | — |

*Route of administration.* — Different routes of administration were compared using a single high dose. Hepatosplenomegaly developed more rapidly after i.v. than after i.p. injection, reaching a maximum 12 days after i.v. and 14 days after i.p. administration. Lewis tumour metastases were equally inhibited by i.v. or i.p. CP. Subcutaneous CP at this dose did not cause hepatosplenomegaly or inhibit the tumour.

### DISCUSSION

Most studies on the effects of CP in experimental animals have used a single high dose of the vaccine; in mouse studies the average dose range has been 75–100 mg/m$^2$ (reviewed by Scott, 1974*a*). The human dose is much lower, 2–10 mg/m$^2$ (Thatcher & Crowther, 1978; Cederholm-Williams *et al.*, 1978; Mitcheson & Castro, 1978) but repeated treatments are often given. At this Institute we give patients with cancer CP at a dose of 10 mg/m$^2$, repeating the treatment at monthly intervals to a total of 6 treatments. This study was designed to determine, in mice, the best treatment regimen for cancer using CP, comparing the traditional mouse dose with a single low, human-equivalent dose (70 $\mu$g) and with repeated human-equivalent doses.

In C57/BL mice a single high dose caused considerable toxic side-effects, including thrombosis (Lampert *et al.*, 1977). A second high dose caused an anaphylactic-type reaction and death (Mitcheson, in preparation). Low doses were much better tolerated, and could be repeated. Both high and low doses caused hepatosplenomegaly, but thrombosis and subsequent infarction and necrosis were much less severe in animals receiving a low dose. Interestingly, repeated doses did not cause fresh pathology.

CP's antitumour action is attributed to non-specific activation of macrophages (reviewed in Milas & Scott, 1978). RES stimulation can be measured by weight increases of liver and spleen or by stimulation of phagocytic activity, and these can be used as indices of anti-tumour activity (Adlam & Scott, 1973; McBride *et al.*, 1975). On this basis we predicted that repeated low doses would give the best anti-tumour effect, but this was not supported by our subsequent findings.

The effect of various single doses of CP on Lewis tumour metastases was determined. The human-equivalent dose was as effective as larger doses and significantly reduced pulmonary metastases. (This excluded the possibility of any summation of effect due to giving sub-optimal doses.) A single low dose was compared with repeated low doses. Both treatments prolonged survival but, again, repeated doses did not confer any extra protection. This supports Scott's observation (1974*b*) that multiple doses of CP were no more effective than a single dose. However, Fisher *et al.* (1975) found that repeated i.p. injections of a therapeutic dose of CP inhibited primary tumour growth more than a single injection and Milas *et al.* (1975) reported that the antitumour protection afforded by repeated sub-optimal doses of CP in mice was greater than that of the total dose given as a single i.v. injection.

CP administered up to 2 weeks before tumour implantation inhibited pulmonary metastasis as effectively as treatment on the day of tumour implantation. Pre-treatment earlier than 2 weeks was ineffective. However, when repeated low-dose prophylaxis was combined with a therapeutic low dose on the day of tumour implantation, the anti-metastatic action was completely neutralized. A similar finding has been reported after repeated prophylaxis with *C. granulosum* (Milas *et al.*, 1975). In contrast, Scott & Warner (1976) found resistance to tumour-cell challenge in mice that had received 14 weekly prophylactic injections of a "human-equivalent" dose of CP (5·25 mg/m$^2$). There are several mechanisms by which the anti-metastatic action of CP could be neutralized:

(1) The RES stimulated by CP may become refractory to further stimulation. This is suggested by the impaired development of hepatosplenomegaly in mice which received a second injection of CP 2 weeks after the first and would explain why repeated low doses did not maintain maximum hepatosplenomegaly. However, this "refractory period" lasts less than 4 weeks after a single injection and neutralization of the anti-metastatic effect still occurred after a 13-week treatment-free interval.

(2) Circulating immune complexes (CIC) can block cell-mediated immunity (Baldwin & Robins, 1976) and large quantities can saturate the RES (Mannik *et al.*, 1974). We have found that repeated doses of CP caused a significant and prolonged increase in CIC in mice (Mitcheson, in preparation).

(3) The high titre of anti-CP antibody produced after repeated doses, or indeed, the injected CP itself, may in some way neutralize the anti-metastatic effect.

(4) CP may promote suppressor-cell activity (Mathé *et al.*, 1978).

Repeated human-equivalent doses of CP in mice have less adverse side-effects and cause most RES change. They raise very high antibody titres and increase circulating immune complexes. However, their anti-tumour effect is no greater than that of a single "human-equivalent" dose and, indeed, repeated prophylaxis neutralizes the protective action. We conclude that in our mouse system the best cancer treatment with CP is a single human-equivalent dose (70 μg) administered i.v. This finding may have clinical implications for *C. parvum* therapy.

The authors would like to thank Dr I. A. Lampert for the histological studies. This investigation was supported by a grant from the Medical Research Council; H. D. Mitcheson was supported by the Wellcome Foundation.

### REFERENCES

ADLAM, C. & SCOTT, M. T. (1973) Lymphoreticular stimulatory properties of *Corynebacterium parvum* and related bacteria. *J. Med. Microbiol.*, **6**, 261.

BALDWIN, R. W. & ROBINS, R. A. (1976) Factors interfering with immunological rejection of tumours. *Br. Med. Bull.*, **32**, 118.

CEDERHOLM-WILLIAMS, S. A., KING, A., ALLINGTON, M. J., GILL, P. G., SHARP, A. A. & BRITTON, B. J. (1978) Coagulation and fibrinolysis during the infusion of *Corynebacterium parvum* in man. *Br. J. Cancer*, **37**, 1074.

FISHER, B., WOLMARK, N., SAFFER, E. & FISHER, E. R. (1975) Inhibitory effect of prolonged *Corynebacterium parvum* and cyclophosphamide administration on the growth of established tumours. *Cancer*, **35**, 134.

HALPERN, B. N., BIOZZI, G., STIFFEL, C. & MOUTON, D. (1966) Inhibition of tumour growth by administration of heat killed *Corynebacterium parvum*. *Nature*, **212**, 853.

HALPERN, B. N., PREVOT, A. R., BIOZZI, G. & 5 others (1963) Stimulation de l'activité phagocytaire du système réticuloendothelial provoquée par *Corynebacterium parvum*. *J. Reticuloendothel. Soc.*, **1**, 77.

ISRAEL, L., EDELSTEIN, R., DEPIERRE, A. & DIMITROV, N. (1975) Brief communication: Daily intravenous infusions with *Corynebacterium parvum* in 20 patients with disseminated cancer: a preliminary report of clinical and biological findings. *J. Natl Cancer Inst.*, **55**, 29.

JAMES, S. E. & SALSBURY, A. J. (1974) Effect of (±) -1, 2-bis (3,5-dioxopiperazin-1-yl) propane on tumour blood vessels and its relationship to the antimetastatic effect in the Lewis lung carcinoma. *Cancer Res.*, **34**, 839.

LAMPERT, I. A., JONES, P. D. E., SADLER, T. E. & CASTRO, J. E. (1977) Intravascular coagulation resulting from intravenous injection of *C. parvum* in mice. *Br. J. Cancer*, **36**, 15.

MANNIK, M., HAAKENSFAD, A. O. & AREND, W. P. (1974) The fate and detection of circulating immune complexes. In: *Progress in immunology II, Vol 5: Clinical aspects II*, Eds. Brent & Holborow. Amsterdam: North-Holland. p. 91.

MATHÉ, G., FLORENTIN, I., OLSSON, L. & 7 others (1978) Pharmacologic factors and manipulation of immunity systemic adjuvants in cancer therapy. *Cancer Treat, Rep.*, **62**, 1613.

MCBRIDE, W. H., DAWES, J., DUNBAR, N., GHAFFER, A. & WOODRUFF, M. F. A. (1975) A comparative study of anaerobic coryneforms. Attempts to correlate their anti-tumour activity with their serological properties and ability to stimulate the lympho-reticular system. *Immunology*, **28**, 49.

MILAS, L. & SCOTT, M. T. (1978) Antitumour activity of *Corynebacterium parvum*. *Adv. Cancer Res.*, **26**, 257.

MILAS, L., BASIC, I., KOGELNIK, H. D. & RODNEY WITHERS, H. (1975) Effects of *Corynebacterium granulosum* on weight and histology of lymphoid organs, response to mitogens, skin allografts, and a syngeneic fibrosarcoma in mice. *Cancer Res.*, **35**, 2365.

MITCHESON, H. D. & CASTRO, J. E. (1978) Clinical studies with *Corynebacterium parvum*. *Dev. Biol. Stand.*, **38**, 509.

PROCTOR, J., RUDENSTAM, C. M. & ALEXANDER, P. (1973) Increased incidence of lung metastases following treatment of rats bearing hepatomas with irradiated tumour cells and the beneficial effect of *Corynebacterium parvum* in this system. *Biomed. Express*, **19**, 248.

SADLER, T. E. & CASTRO, J. E. (1976) The effects of *Corynebacterium parvum* and surgery on the Lewis lung carcinoma and its metastases. *Br. J. Surg.*, **63**, 292.

SARNA, G. P., LOWITZ, B. B., HASKELL, C. M. & CLINE, M. J. (1977) Chemo-immunotherapy of bronchogenic carcinoma. *Proc. Am. Assoc. Cancer Res.*, **18**, 89.

SCOTT, M. T. (1974*a*) *Corynebacterium parvum* as an immunotherapeutic anticancer agent. *Seminars Oncol.*, **1**, 367.

SCOTT, M. T. (1974*b*) *Corynebacterium parvum* as a therapeutic antitumour agent in mice. I. Systemic effects from intravenous injection. *J. Natl Cancer Inst.*, **53**, 855.

SCOTT, M. T. & WARNER, S. L. (1976) The accumulated effects of repeated systemic or local injections of low doses of *Corynebacterium parvum* in mice. *Cancer Res.*, **36**, 1335.

SIMPSON-HERREN, L., SANFORD, A. H. & HOLMQUIST, J. P. (1974) Cell population kinetics of transplanted and metastatic Lewis lung carcinoma. *Cell Tiss. Kinet.*, **7**, 349.

SMITH, S. E. & SCOTT, M. T. (1972) Biological effects of *Corynebacterium parvum*. III. Amplification of resistance and impairment of active immunity to murine tumours. *Br. J. Cancer*, **26**, 361.

SUGIURA, K. & STOCK, C. C. (1955) Studies in a tumour spectrum: II. The effect of phosphoramides on the growth of a variety of mouse and rat tumours. *Cancer Res.*, **15**, 38.

TAKITA, H. & MOAYERI, H. (1976) Effects of *Corynebacterium parvum* and chemotherapy in lung carcinoma. *Proc. Am. Soc. Clin. Oncol.*, **17**, 292.

THATCHER, N. & CROWTHER, D. (1978) Effects of BCG and *Corynebacterium parvum* on immune reactivity in melanoma patients. *Dev. Biol. Stand.*, **38**, 449.

WEXLER, H. (1966) Accurate identification of experimental pulmonary metastases. *J. Natl Cancer Inst.*, **36**, 641.

WOODRUFF, M. F. A. & BOAK, J. L. (1966) Inhibitory effect of injection of *Corynebacterium parvum* on the growth of tumour transplants in isogeneic hosts. *Br. J. Cancer*, **20**, 345.


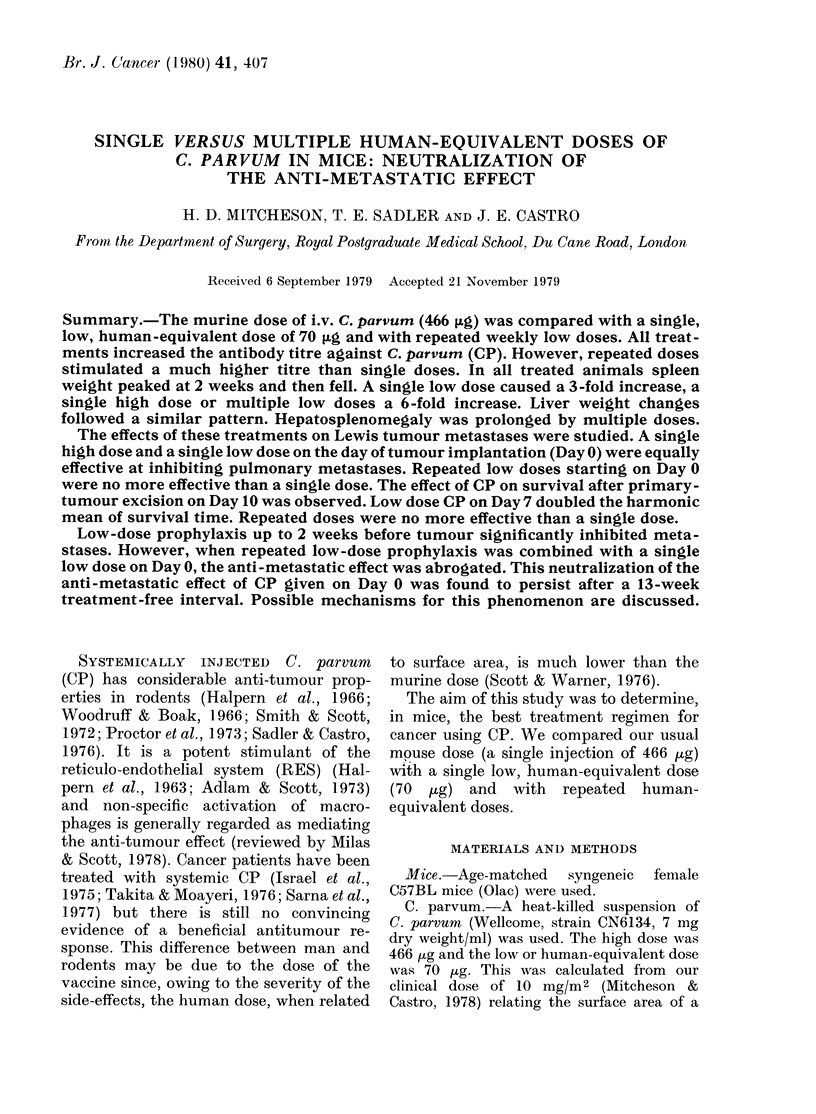

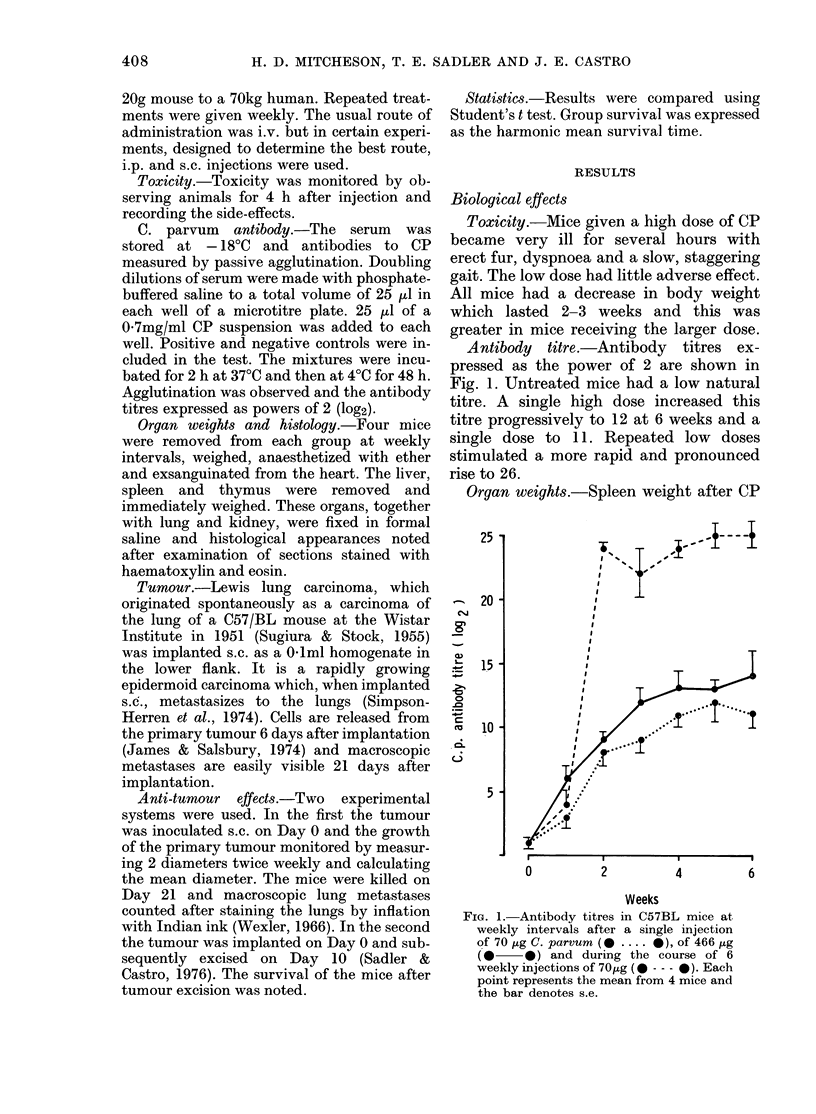

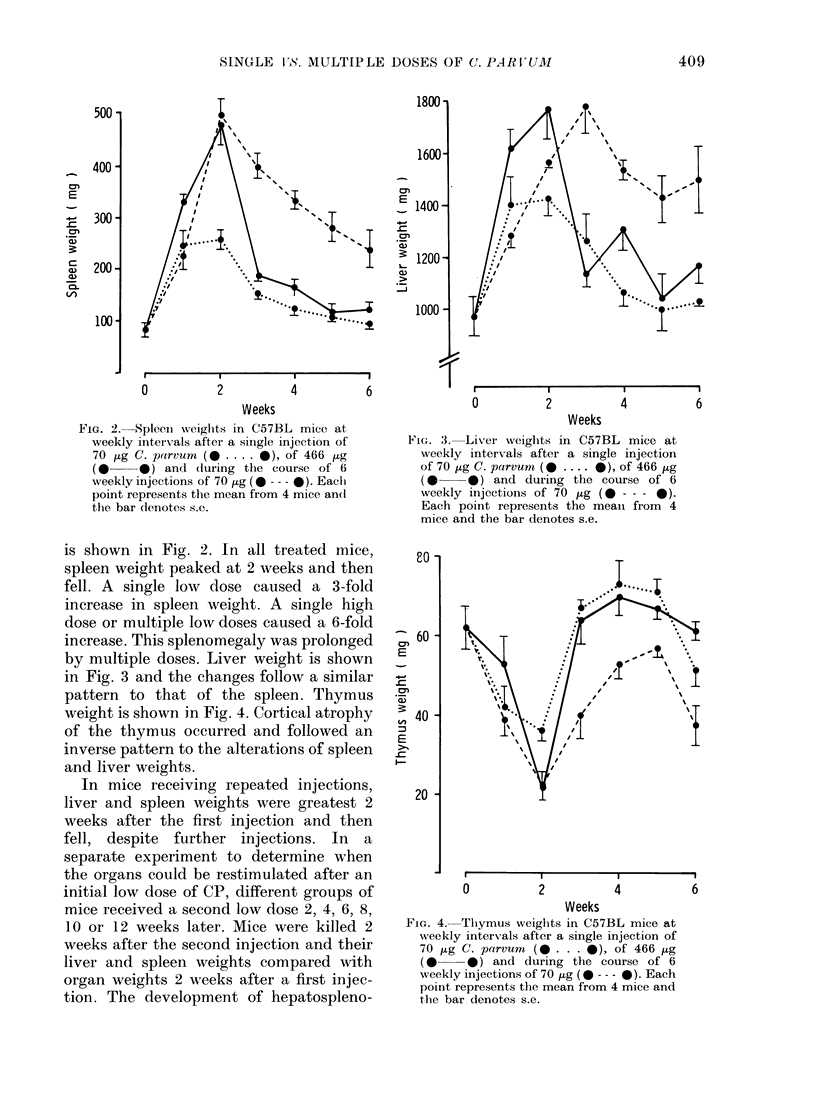

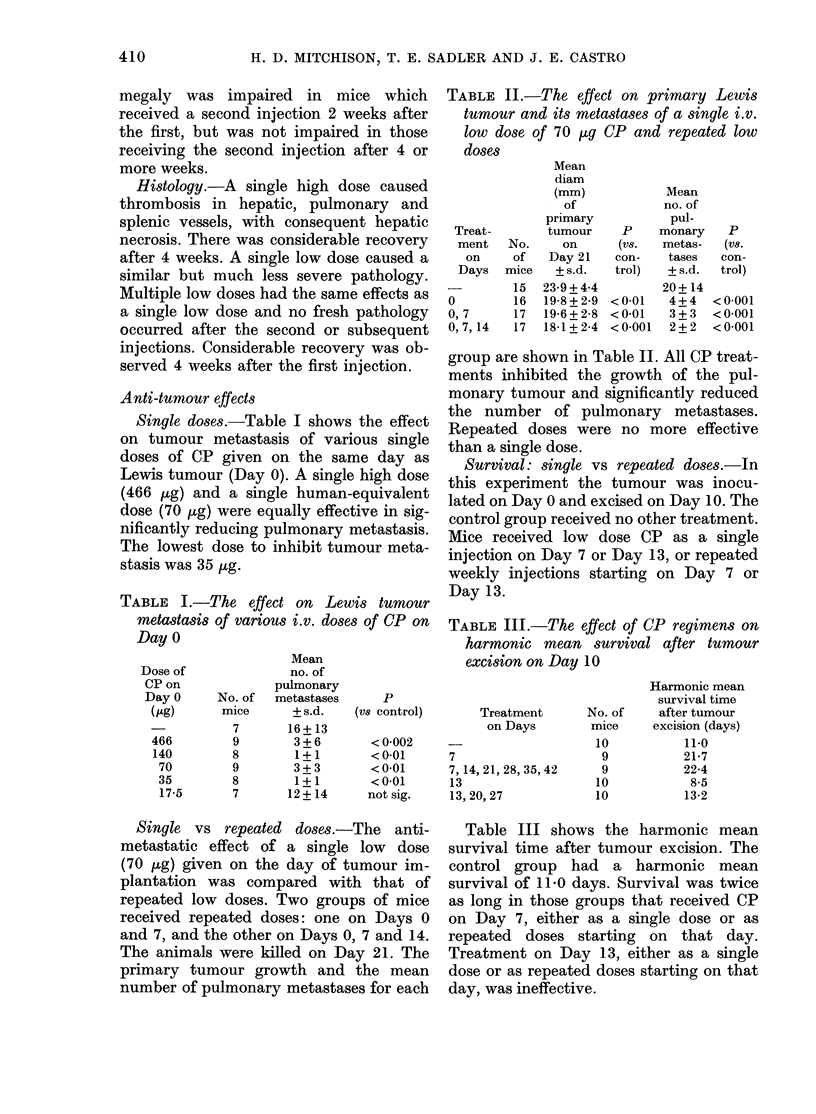

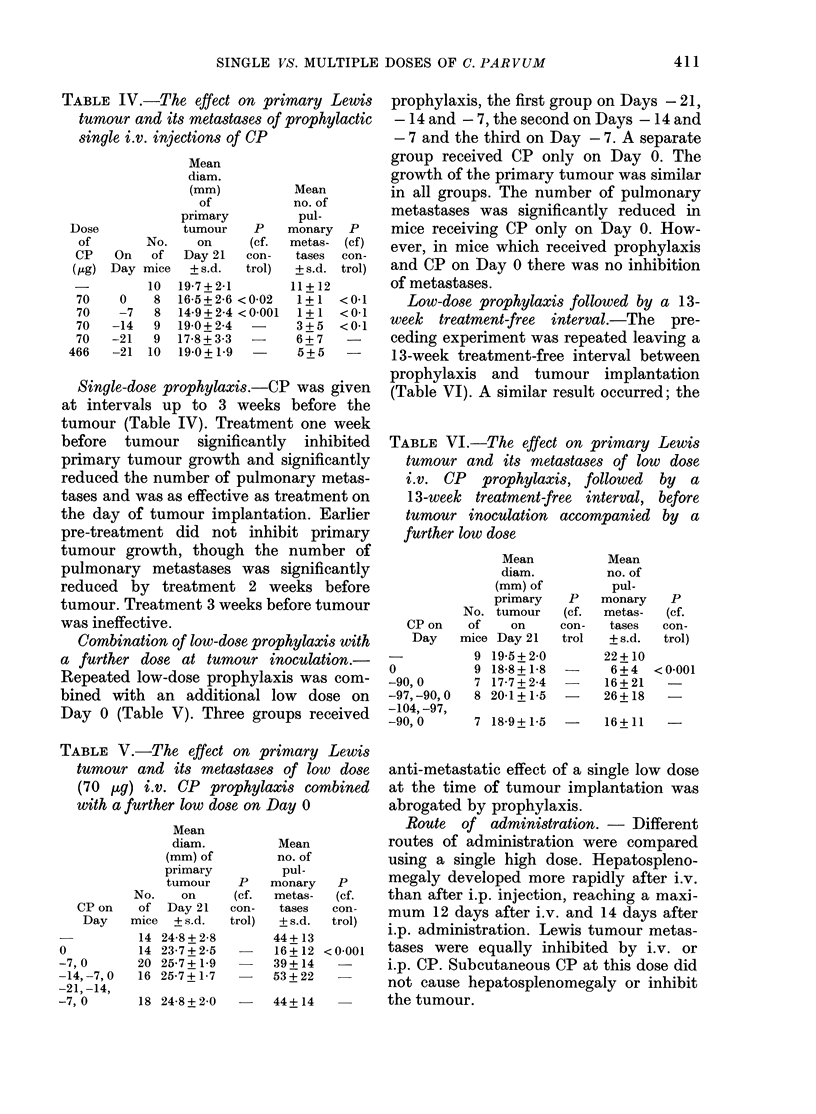

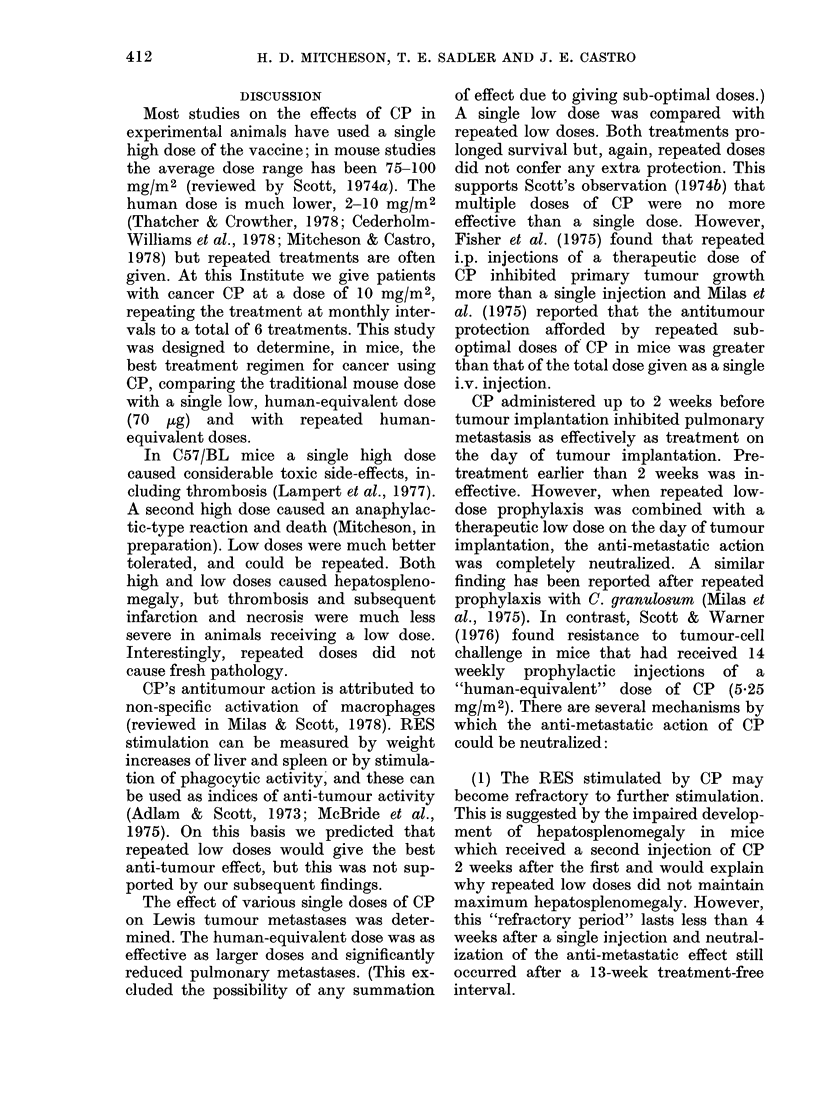

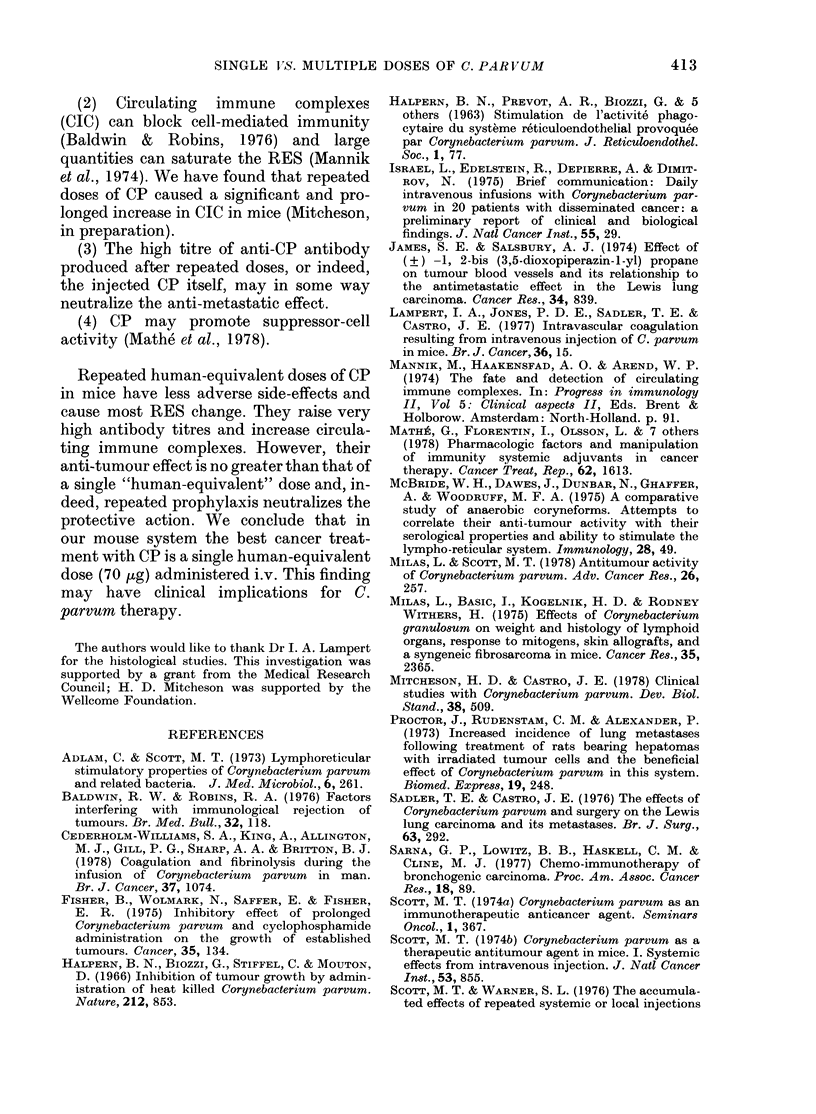

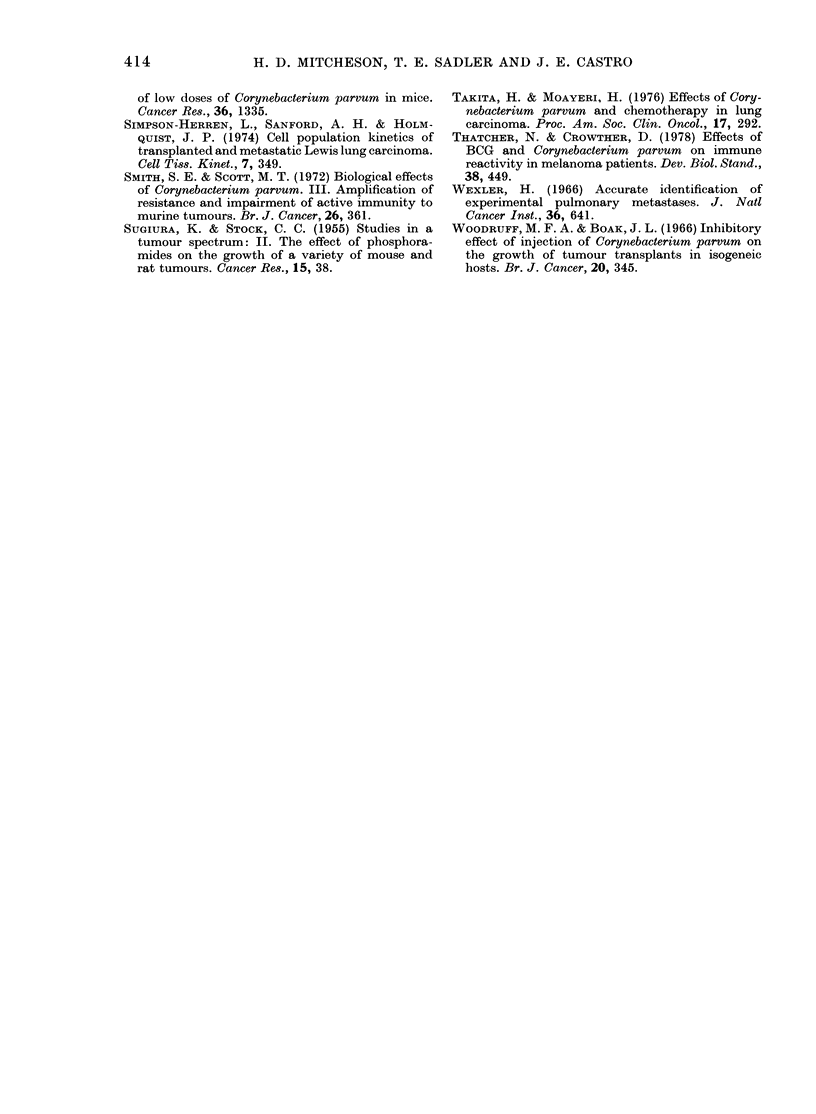


## References

[OCR_00965] Adlam C., Scott M. T. (1973). Lympho-reticular stimulatory properties of Corynebacterium parvum and related bacteria.. J Med Microbiol.

[OCR_00970] Baldwin R. W., Robins R. A. (1976). Factors interfering with immunological rejection of tumours.. Br Med Bull.

[OCR_00975] Cederholm-Williams S. A., King A., Allington M. J., Gill P. G., Sharp A. A., Britton B. J. (1978). Coagulation and fibrinolysis during the infusion of Corynebacterium parvum in man.. Br J Cancer.

[OCR_00982] Fisher B., Wolmark N., Saffer E., Fisher E. R. (1975). Inhibitory effect of prolonged Corynebacterium parvum and cyclophosphamide administration on the growth of established tumors.. Cancer.

[OCR_00989] Halpern B. N., Biozzi G., Stiffel C., Mouton D. (1966). Inhibition of tumour growth by administration of killed corynebacterium parvum.. Nature.

[OCR_01004] Israël L., Edelstein R., Depierre A., Dimitrov N. (1975). Daily intravenous infusions of Corynebacterium parvum in twenty patients with disseminated cancer: a preliminary report of clinical and biologic findings.. J Natl Cancer Inst.

[OCR_01010] James S. E., Salsbury A. J. (1974). Effect of (plus or minus)-1,2-bis(3,5-dioxopiperazin-1-yl)propane on tumor blood vessels and its relationship to the antimetastatic effect in the Lewis lung carcinoma.. Cancer Res.

[OCR_01017] Lampert I. A., Jones P. D., Sadler T. E., Castro J. E. (1977). Intravascular coagulation resulting from intravenous injection of C. parvum in mice.. Br J Cancer.

[OCR_01030] Mathé G., Florentin I., Olsson L., Bruley-Rosset M., Schulz J., Kiger N., Orbach-Arbouys S., Schwarzenberg L., Pouillart P., de Vassal F. (1978). Pharmacologic factors and manipulation of immunity systemic adjuvants in cancer therapy.. Cancer Treat Rep.

[OCR_01036] McBride W. H., Dawes J., Dunbar N., Ghaffar A., Woodruff M. F. (1975). A comparative study of anaerobic Coryneforms. Attempts to correlate their anti-tumour activity with their serological properties and ability to stimulate the lymphoreticular system.. Immunology.

[OCR_01051] Milas L., Basic I., Kogelnik H. D., Withers H. R. (1975). Effects of Corynebacterium granulosum on weight and histology of lymphoid organs, response to mitogens, skin allografts, and a syngeneic fibrosarcoma in mice.. Cancer Res.

[OCR_01044] Milas L., Scott M. T. (1978). Antitumor activity of Corynebacterium parvum.. Adv Cancer Res.

[OCR_01057] Mitcheson H. D., Castro J. E. (1977). Clinical studies with Corynebacterium parvum.. Dev Biol Stand.

[OCR_01062] Proctor J., Rudenstam C. M., Alexander P. (1973). Increased incidence of lung metastases following treatment of rats bearing hepatomas with irradiated tumour cells and the benefical effect of Corynebacterium parvum in this system.. Biomedicine.

[OCR_01114] SUGIURA K., STOCK C. C. (1955). Studies in a tumor spectrum. III. The effect of phosphoramides on the growth of a variety of mouse and rat tumors.. Cancer Res.

[OCR_01070] Sadler T. E., Castro J. E. (1976). The effects of Corynebacterium parvum and surgery on the Lewis lung carcinoma and its metastases.. Br J Surg.

[OCR_01087] Scott M. T. (1974). Corynebacterium parvum as a therapeutic antitumor agent in mice. I. Systemic effects from intravenous injection.. J Natl Cancer Inst.

[OCR_01082] Scott M. T. (1974). Corynebacterium parvum as an immunotherapeutic anticancer agent.. Semin Oncol.

[OCR_01093] Scott M. T., Warner S. L. (1976). The accumulated effects of repeated systemic or local injections of low doses of Corynebacterium parvum in mice.. Cancer Res.

[OCR_01108] Smith S. E., Scott M. T. (1972). Biological effects of Corynebacterium parvum. 3. Amplification of resistance and impairment of active immunity to murine tumours.. Br J Cancer.

[OCR_01124] Thatcher N., Crowther D. (1977). Effects of BCG and Corynebacterium parvum on immune reactivity in melanoma patients.. Dev Biol Stand.

[OCR_01130] Wexler H. (1966). Accurate identification of experimental pulmonary metastases.. J Natl Cancer Inst.

[OCR_01135] Woodruff M. F., Boak J. L. (1966). Inhibitory effect of injection of Corynebacterium parvum on the growth of tumour transplants in isogenic hosts.. Br J Cancer.

